# Metabolomic datasets in COVID-19 research: a systematic literature review of availability, characteristics, and methodologies

**DOI:** 10.7717/peerj.21313

**Published:** 2026-07-09

**Authors:** Hugo A. Torres-Pasillas, Huizilopoztli Luna-García, José María Celaya-Padilla, Carlos H. Espino-Salinas, Claudia Acra-Despradel, Klinge Villalba-Condori, Yenhny Margareth Cárdenas Núñez

**Affiliations:** 1 Unidad Académica de Ingeniería Eléctrica, Universidad Autónoma de Zacatecas, Zacatecas, Mexico; 2 Universidad Nacional Pedro Henríquez Ureña, Santo Domingo, Dominican Republic; 3 Universidad Católica de Santa María, Arequipa, Peru

**Keywords:** COVID-19, Metabolomic datasets, Metabolomics, Data sharing, FAIR, Systematic review, Biomarkers

## Abstract

The COVID-19 pandemic has accelerated the integration of metabolomics and Machine Learning in biomedical research, resulting in the creation of numerous datasets with high potential for reuse. However, information regarding their accessibility, quality, and usability remains scattered and inconsistent. This systematic review aims to identify and evaluate publicly available human metabolomic datasets related to COVID-19, providing detailed information on their main characteristics and how to access them, with the goal to inform their potential for reuse in future research. Following PRISMA guidelines and the Kitchenham methodology, we conducted a comprehensive search of the scientific literature and specialized metabolomics repositories, identifying 110 unique datasets. Each dataset was assessed based on 15 variables related to data availability, accessibility, collection methodologies, sample sizes, and the extent of participant metadata provided. These datasets offer significant value for secondary analyses and ML applications, contributing to insights into disease mechanisms, early diagnosis, and patient stratification. By offering a structured overview of dataset characteristics, this review aims to support researchers in identifying suitable resources, encourages data reuse, and promotes best practices for data sharing and standardization in the context of COVID-19 and metabolomics. Nonetheless, our findings reveal critical limitations, including the underuse of dedicated repositories, frequent unavailability of raw data, lack of standardization in processed data, and insufficient metadata—particularly regarding participant demographics and clinical information. Inconsistencies in data formats and reporting standards further hinder dataset findability, interoperability, and reuse. To enhance the value and impact of future metabolomic research, we recommend adopting standardized reporting guidelines, improving metadata completeness, ensuring the availability of raw data, and promoting the use of interoperable repositories to facilitate reproducibility, integration, and broader application of shared datasets.

## Introduction

Since the World Health Organization (WHO) declared COVID-19 a global pandemic in March 2020 (Cucinotta & Vanelli, 2020), the scientific community has produced an unprecedented volume of research, generating over 480,000 publications to date (Chen et al., 2023). This global response has led to the creation of extensive datasets across academic, governmental, and private sectors (Zuo et al., 2021), accelerating the integration of artificial intelligence (AI) and data science in healthcare (Wang et al., 2021). Among the omics disciplines, metabolomics has emerged as a powerful approach for investigating COVID-19 pathophysiology and the host’s biochemical response to infection.

Metabolomics involves the comprehensive analysis of small-molecule metabolites that represent the functional end-products of cellular processes (Kell & Oliver, 2016). Unlike genomics, which provides information on static susceptibility and potential risk factors, or proteomics and transcriptomics, which elucidate the machinery of cellular response, metabolomics captures the ultimate phenotypic outcome of these interactions (Kell & Oliver, 2016; Idle & Gonzalez, 2007). This distinction is particularly critical in COVID-19 research, where clinical manifestations vary drastically among individuals with similar genetic backgrounds. While genomic studies have identified host factors associated with severity, they cannot account for the dynamic, real-time metabolic alterations driven by the viral infection, environmental exposures, and comorbidities. Metabolomics provides a snapshot of these complex interactions, revealing immediate disturbances in energy metabolism, immune regulation, and lipid signaling that other omics layers might miss or detect only indirectly (Idle & Gonzalez, 2007; Klassen et al., 2017). Consequently, it is uniquely positioned to identify disease-related changes and potential therapeutic targets with high sensitivity (Hasan, Suleiman & Perez-Lopez, 2021).

The integration of machine learning (ML) with metabolomics has gained momentum for its ability to detect complex patterns in high-dimensional biochemical data (Wishart, 2016). ML algorithms can assist in biomarker discovery, patient stratification, and the development of predictive models for diagnosis and prognosis (Blaženović et al., 2018; Xu et al., 2024). This approach has shown potential in various diseases, including cancer (Seyfried & Shelton, 2010; Brown et al., 2012; Chen et al., 2024; Kuwabara et al., 2022), diabetes (Leiherer et al., 2024; Hirakawa et al., 2022; Zhang & Yang, 2022), and more recently, COVID-19.

In the context of COVID-19, several studies have developed metabolomics-based ML models to classify disease severity, predict outcomes, and support clinical decision-making (Shen et al., 2020; Baiges-Gaya et al., 2023). For instance, Shen et al. (2020) identified lipid alterations and used random forest models to classify severe cases, while Baiges-Gaya et al. (2023) used gradient boosting machines to differentiate between COVID-19 patients and healthy controls based on serum metabolite profiles. Other works have demonstrated the feasibility of using such approaches for early diagnosis and severity prediction (López-Hernández et al., 2021; Thomas et al., 2020; Wu et al., 2020; Sumon et al., 2025; Robinson et al., 2025), reinforcing the potential of ML-enhanced metabolomics to advance precision medicine in COVID-19 care (Hasan, Suleiman & Perez-Lopez, 2021; Bardanzellu & Fanos, 2022).

Despite this promise, the application of ML to metabolomics faces specific computational and methodological challenges. A primary hurdle is the “curse of dimensionality”, where the number of detected metabolic features (*p*) vastly outnumbers the available samples (*n*). This imbalance makes ML models highly prone to overfitting, capturing noise instead of true biological signals, especially in small-scale COVID-19 cohorts (Liebal et al., 2020; Broadhurst et al., 2018). Additionally, technical sources of variation—such as batch effects and inter-instrument differences—can introduce non-biological bias, leading algorithms to learn site-specific patterns rather than generalizable disease markers (Kumar & Misra, 2019). Data sparsity is another critical issue; metabolomics datasets often contain high rates of missing values (*e.g.*, due to limits of detection), which require specialized imputation strategies distinct from standard tabular data approaches (Wei et al., 2018). Finally, the lack of standardized metadata formats limits the ability to merge datasets for validating models across different populations, hindering the development of robust, clinically deployable tools (Shuja et al., 2021).

To overcome these limitations, data sharing and the adoption of best practices are critical. One of the main obstacles to training reliable ML models is the limited availability of high-quality, standardized datasets (Shuja et al., 2021). Given the high cost and effort associated with generating metabolomics data, promoting open data access enhances the value of existing resources, reduces sampling bias, and supports model generalization.

Efforts to improve data reusability are supported by frameworks such as Findable, Accessible, Interoperable, and Reusable (FAIR), which provides principles to ensure that digital assets are discoverable and usable by both humans and machines (Boeckhout, Zielhuis & Bredenoord, 2018). These are complemented by the Transparency, Responsibility, User focus, Sustainability, and Technology (TRUST) principles, which define the requirements for digital repositories to maintain data integrity and long-term stewardship (Lin et al., 2020). Furthermore, domain-specific metadata standards from the Metabolomics Standards Initiative (MSI) establish the minimum information necessary to describe metabolomics experiments, ensuring that chemical and biological contexts are preserved (Fiehn et al., 2007). Public repositories like MetaboLights (Yurekten et al., 2024) and the Metabolomics Workbench (Sud et al., 2016) have been instrumental in implementing these principles, offering platforms for standardized data deposition, analysis tools, and educational resources. Furthermore, the increasing use of data sharing statements in publications fosters transparency by requiring authors to explicitly state how and where their data can be accessed (Taichman et al., 2017; Federer et al., 2018). These practices are essential to enhance reproducibility and collaboration in metabolomics research.

Although previous reviews have addressed data sharing in the context of COVID-19, they have predominantly focused on general open-data landscapes without specific attention to the complexities of omics data (Zuo et al., 2021; Shuja et al., 2021). In the specific domain of metabolomics, early efforts such as the meta-analysis by Pang et al. (2021) provided crucial insights into the biological pathways altered by the infection. However, that study was limited to the initial phase of the pandemic and focused primarily on aggregating biological findings rather than evaluating the reusability and accessibility of the underlying raw datasets. To date, there is no systematic assessment of the vast amount of metabolomics data generated in subsequent years, nor a detailed evaluation of their metadata quality and readiness for secondary analysis, particularly for data-driven approaches like Machine Learning.

In this context, this systematic review aims to enhance data reuse in COVID-19 metabolomics by compiling and evaluating a comprehensive collection of publicly available human metabolomic datasets, with a particular focus on those with potential for ML-driven analyses. We examine essential characteristics—including accessibility, metadata completeness, methodologies, and participant information—to facilitate their integration into robust computational models.

The key research questions guiding this review are:

• **Availability and accessibility:** Which metabolomics datasets related to COVID-19 are publicly available, through which repositories or platforms, and in which formats are they shared?
• **Data collection and characteristics:** What methodologies were employed for data collection, and what are the key characteristics of the datasets, including sample types, metabolomics platforms, and dataset size?
• **Participant metadata and classification factors:** What additional participant data are included in these datasets, and what variables are used for classification purposes?

By addressing these questions, this review will provide valuable insights into the current landscape of COVID-19 metabolomics data sharing, identify gaps and challenges, and offer recommendations for improving the availability and usability of such datasets. Ultimately, the work aims to support the development of metabolomics-based tools for COVID-19 diagnosis, prognosis, and precision medicine, providing a reliable, well-documented resource of available datasets. Moreover, it seeks to foster a culture of open science and collaboration in metabolomics research, facilitating future studies that can leverage these datasets for broader biomedical advancements.

## Materials and methods

**Figure 1:**
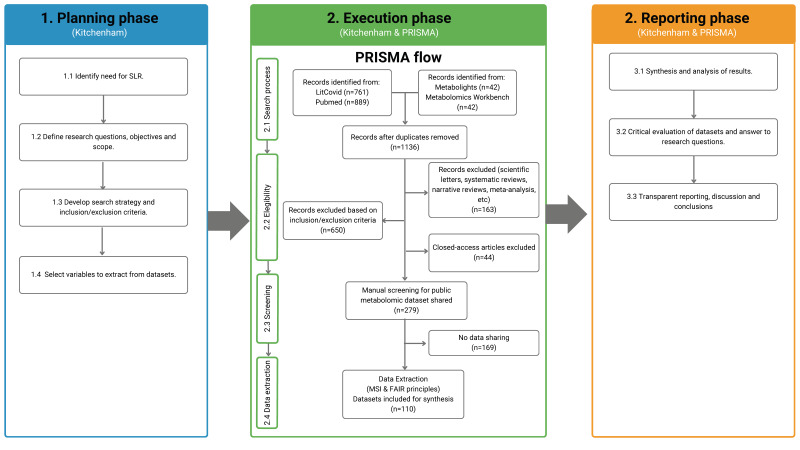
Methodological framework of the systematic literature review, integrating the phases proposed by Kitchenham (planning, execution, and reporting) with the PRISMA flow diagram.

To conduct this systematic literature review (SLR), we followed the guidelines proposed by Kitchenham (2004), structured into three key phases: planning, execution, and reporting. We complemented this approach with the PRISMA framework (Moher et al., 2009), which was particularly applied during the execution and reporting phases to standardize study identification, screening, eligibility assessment, and final inclusion. No protocol was registered prior to conducting the review. The complete methodology is summarized and visually represented in Fig. 1.

 In the planning phase, we established the necessity of conducting a systematic review and clearly defined the research questions, objectives, and scope. This need arises from the fact that, although several metabolomics datasets related to COVID-19 have been made publicly available, their documentation regarding characteristics, quality, and research relevance is often incomplete or inconsistent. Such lack of standardization poses significant challenges for data reuse and cross-study integration. Therefore, this review seeks to bridge this gap by systematically identifying, categorizing, and evaluating available datasets in a structured manner.

During the execution phase, a comprehensive search was performed across selected scientific databases and metabolomics-specific data-sharing platforms. Data extraction aligned with the MSI recommendations and FAIR principles. The final reporting phase synthesizes these findings to ensure reproducibility and utility for the scientific community.

To ensure a methodologically sound and reliable review, the selection of variables to extract from each dataset was guided by both their relevance to current research needs and the standards established by the MSI. The MSI emphasizes the importance of reporting comprehensive metadata, including sample type, analytical methods, preprocessing steps, and contextual clinical information, to enhance data transparency, interoperability, and reusability. Consequently, this review focused on extracting standardized information such as sample origin (*e.g.*, serum, plasma, urine), analytical platform (*e.g.*, Liquid Chromatography-Mass Spectrometry (LC-MS), Gas Chromatography-Mass Spectrometry (GC-MS), Nuclear Magnetic Resonance (NMR)), cohort details (*e.g.*, number of participants, age, sex, disease severity), availability of clinical and experimental metadata, and data preprocessing status.

### Inclusion criteria

• Studies utilizing metabolomic techniques for the analysis of human biological samples in the context of COVID-19.
• Publications published between December 2019 and March 2026.

### Exclusion criteria

• Studies conducted on animal models or cell cultures, or those not employing metabolomics.
• Multi-omics investigations with a limited focus on metabolomics.
• Reviews, commentaries, or studies lacking experimental data.
• Publications without full-text access.

### Scientific literature search strategy

For the identification of scientific articles we searched the following sources: LitCovid (National Library of Medicine, USA), a literature database dedicated to COVID-19 research articles; PubMed, a free resource for biomedical and life sciences literature; and two specialized metabolomics repositories, Metabolomics Workbench and MetaboLights.

These sources were selected to ensure a comprehensive and complementary search strategy, combining curated biomedical literature databases (LitCovid and PubMed) with the two major public repositories that archive metabolomics datasets with standardized metadata (Metabolomics Workbench and MetaboLights).

All sources were last searched on March 13, 2026. The following search terms were used:

• **PubMed:** (“covid-19” OR “sars-cov-2”) AND (“metabolomics” OR “metabolic profiling” OR “metabolite analysis” OR “metabolic biomarkers”). This search returned a total of 889 publications.
• **LitCovid:** (“metabolomics” OR “metabolic profiling” OR “metabolic analysis” OR “metabolic biomarkers”), yielding a total of 761 results.
• **Metabolomics Repositories:** MetaboLights and Metabolomics Workbench were queried using the search terms “COVID-19” and “SARS-CoV-2”, resulting in 42 unique results each of them.

The search and identification process is illustrated in Fig. 1. After removing duplicates, 1,136 unique publications were identified. Hugo A. Torres-Pasillas manually screened these records against eligibility criteria, with independent verification by José M. Celaya Padilla and Huizilopoztli Luna-García. Reviewers worked independently during verification, and no automation tools were used in the process. We excluded 163 irrelevant publication types (*e.g.*, letters, reviews) and 650 articles that did not meet inclusion criteria. Furthermore, 44 articles that were not open access were excluded, as lack of access to the full text prevented identification of shared datasets and/or their characteristics. As a result, a final set of 279 articles was obtained.

### Identification of datasets

Each of the 279 identified articles was manually reviewed to determine whether the authors shared their original dataset. In general, data availability is addressed in a Data Availability Statement (DAS), a similarly named section, or in the Supplementary Materials of the article. When data availability was not explicitly stated and the data were not found in the Supplementary Materials the full text was reviewed to identify any references to data sharing or access.

In total, 239 articles included a DAS. Of these, 114 mentioned the availability of their data—either through a link, an online repository identifier, or in the Supplementary Materials. However, when attempting to access these datasets, 18 were unavailable due to broken links, incorrect dataset, or absence from the repository, resulting in 96 datasets found.

On the other hand, 14 articles did not include a DAS but still shared their metabolomics data *via* Supplementary Materials (*n* = 9), Zenodo (*n* = 1), or MetaboLights (*n* = 4).

As a result, 110 articles included publicly accessible datasets. Notably, two of these articles analyzed data from the same clinical cohort (COMETA) but represent distinct data releases (initial study ST002087; expanded study ST002404 with 605 subjects). Additionally, 82 articles stated in their DAS that the data could be obtained from the authors upon reasonable request, while the remainder lacked a DAS, provided unclear information, or explicitly stated that data were unavailable due to ethical restrictions or privacy concerns (*e.g.*, patient confidentiality), or reserved for future analysis.

### Data extraction strategy

Each of the 110 articles/datasets was manually reviewed by Hugo A. Torres-Pasillas and subsequently verified by Jose M. Celaya Padilla to ensure accuracy and completeness. Any discrepancies between the primary extractor and the verifier were resolved through discussion and consensus. No automation tools were used, and no additional data were requested from study investigators. The variables were determined based on information found in the methodology and data availability sections, as well as through examination of the shared datasets, their metadata, and the Supplementary Files of the articles. In total, 15 descriptive variables were evaluated and grouped into three main categories:

• **Data availability and accessibility:** (1) repository used, (2) availability of processed data (including annotated matrices), (3) availability of raw data, and (4) provided data formats.
• **Study design and collection details:** (5) geographic region, (6) collection period, (7) biological sample type, (8) metabolomics platform, (9) analytical approach, (10) total identified metabolites, and (11) study sample size.
• **Participant classification and metadata:** (12) demographic data availability, (13) clinical symptoms availability, (14) comorbidities information availability, and (15) patient group classification criteria.

Due to the heterogeneity observed in variable 10, which arose from differences in data-sharing practices, the number of variables reported in each article—when available—was used as a reference. If the article did not explicitly state this number, it was estimated by counting the distinct compounds identified in the provided datasets. These discrepancies were often caused by the exclusion of metabolites with high levels of missing data in some studies, as well as by the duplication of metabolites within datasets due to the use of different ionization methods or analytical platforms.

### COVID-19 metabolomics dataset analysis

Descriptive analyses of the 15 extracted variables were conducted to summarize dataset characteristics of the included datasets. Tabular presentations and graphical representations were used to illustrate trends, distributions, and frequencies across the datasets. No data imputation was performed; instead, missing information was excluded from the descriptive statistics and explicitly noted when relevant.

## Results

### Q1. Availability and accessibility

Based on the reviewed literature, a total of 110 metabolomics datasets related to COVID-19, listed in Table S1, were identified, spanning from December 2019 to March 2026. Of these, 65 were deposited in public data repositories, while 47 were made available as Supplementary Materials in scientific publications. Notably, two datasets from Juyal et al. (2026) and Gardinassi et al. (2023) were found both in the Supplementary Materials and through a external repository, and therefore were counted twice.

#### Data host

**Figure 2:**
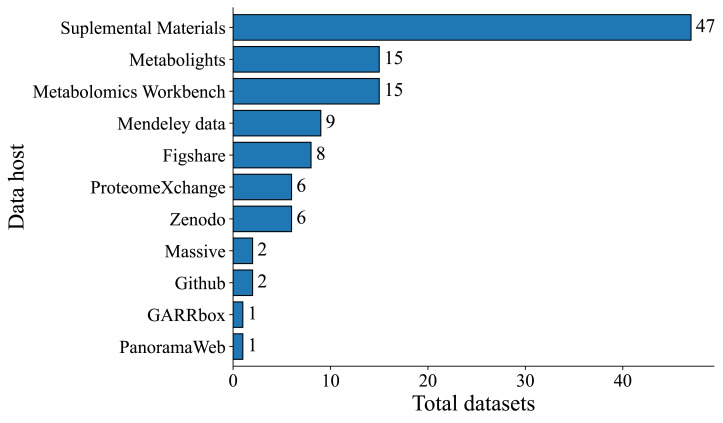
Total datasets stored in each data-sharing repository. Two datasets were found both in Supplementary Materials and within a repository, and therefore are counted twice.

Figure 2 details the distribution of platforms used to share the datasets. Most frequently, data were provided as journal Supplementary Materials, a method that often lacks the structured metadata, persistent identifiers, and standardization needed for long-term interoperability, followed by specialized repositories such as Metabolomics Workbench and MetaboLights, which are recognized for their better adherence to MSI recommendations, ensuring comprehensive experimental documentation. The remaining datasets were distributed across various general-purpose repositories and individual platforms.

#### Data format

**Figure 3:**
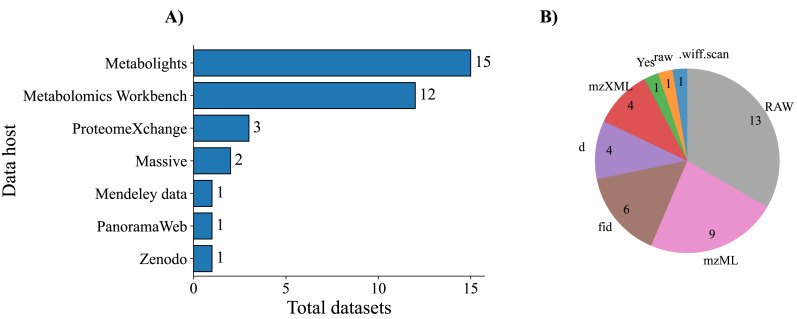
Distribution of metabolomics datasets shared as raw data in COVID-19 research. (A) Number of datasets hosted by each repository. (B) Distribution of raw data file formats.

Among the shared datasets, 39 provided raw data. As detailed in Fig. 3A, metabolomics-specialized repositories were the most common hosts for raw datasets. Additionally, platforms primarily designed for proteomics, such as ProteomeXChange and MassIVE, were also used, particularly in studies involving multi-omics analyses (He et al., 2021; Li et al., 2022) or focused exclusively on metabolomics (Li et al., 2023; Tahir et al., 2024; Sameh et al., 2023; Overmyer et al., 2021). Other datasets were shared through PanoramaWeb (Richard et al., 2022) and the general-purpose repository Mendeley Data (Marhuenda-Egea et al., 2023).

Figure 3B illustrates the file formats used, comprising both proprietary vendor-specific extensions (*e.g.*, .RAW, .fid, .d) and open community standards (*e.g.*, mzML, mzXML). While open formats favor long-term interoperability, raw data reuse also heavily depends on comprehensive experimental metadata. Despite these recognized benefits, only about one-third of the reviewed datasets included raw files, often due to practical challenges like large file sizes. Bridging this data-sharing gap requires broader adoption of standardized open formats and robust metadata reporting in stable repositories.

**Figure 4:**
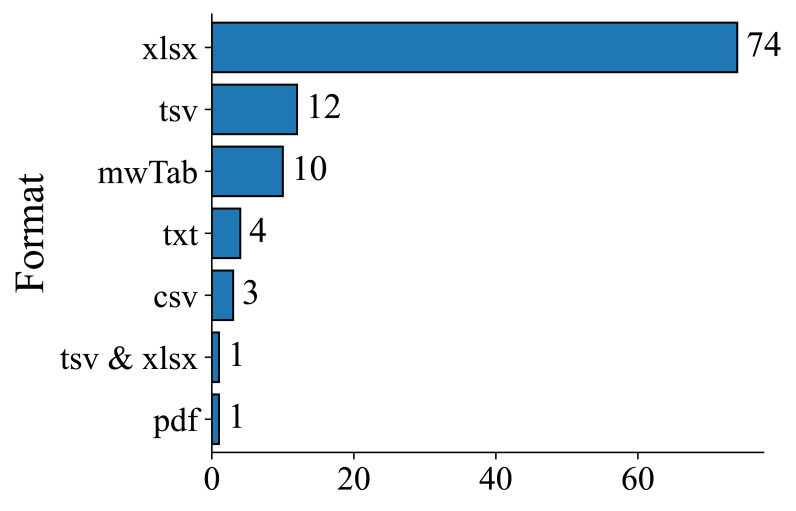
Distribution of file formats used for sharing processed metabolomics datasets (*n* = 105).

On the other side, a total of 105 studies shared their processed metabolomics datasets in a variety of file formats (Fig. 4), containing metabolite abundance matrices and occasionally accompanied by participant demographics, clinical metadata or statistical analysis outputs.

In general, researchers predominantly favored spreadsheet-based formats (*e.g.*, .xlsx) for their accessibility and human readability. However, these formats often pose challenges for automated processing and cross-study integration. Conversely, the use of structured, machine-readable formats like mwTab reflects a growing adherence to MSI best practices, which strongly recommend standardized formats and comprehensive metadata to ensure long-term data interoperability and reusability.

### Q2. Study design and dataset characteristics

#### Biological sample

**Figure 5:**
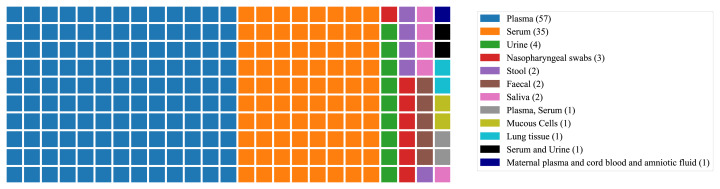
Distribution of biological sample types used in the shared COVID-19 metabolomics datasets.

As shown in Fig. 5, plasma (*n* = 57) and serum (*n* = 35) were the most commonly analyzed biological matrices. Other less frequent sample types—often tailored to specific research objectives—included urine, feces, lung tissue, mucosal cells, saliva, nasopharyngeal swabs, and perinatal fluids. Additionally, some studies incorporated multiple matrices for broader metabolome characterization, such as combining serum and urine (Bi et al., 2022) or investigating various maternal fluids during pregnancy (Frankevich et al., 2023).

#### Metabolomics platforms

**Figure 6:**
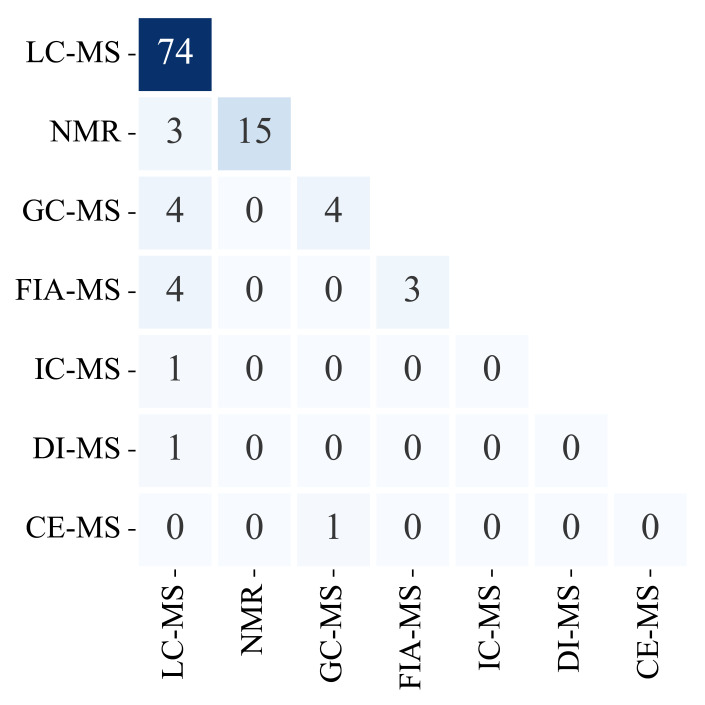
Metabolomics platforms used in the analyzed datasets. Diagonal values correspond to studies using a single platform; off-diagonal values indicate combinations.

The choice of metabolomics platform impacts metabolite coverage and cross-study comparability. As illustrated in the co-occurrence matrix (Fig. 6), the analyzed datasets employed diverse analytical strategies. LC-MS was the predominant platform, utilized either independently or combined with techniques like GC-MS, Flow Injection Analysis-Mass Spectrometry (FIA-MS), or NMR. Standalone applications of NMR, GC-MS, or Capillary Electrophoresis-Mass Spectrometry (CE-MS) were also observed, though less frequently. This diversity highlights both the technological range in metabolomics research and the need for clear methodological reporting to support data reuse and interpretation.

Further methodological details revealed notable heterogeneity within platform categories. Among mass spectrometry-based studies, 62 utilized tandem mass spectrometry (MS/MS). Liquid chromatography techniques also varied widely, encompassing ultra-performance liquid chromatography (UPLC, *n* = 39), ultra-high-performance liquid chromatography (UHPLC, *n* = 14), high-performance liquid chromatography (HPLC, *n* = 3), and nanoflow LC (nanoLC, *n* = 1). Furthermore, several datasets specified advanced instrumentation configurations, including quadrupole time-of-flight (QTOF, *n* = 8), high-resolution mass spectrometry (HRMS, *n* = 9), and multiple reaction monitoring (MRM, *n* = 2).

#### Number of participants

**Figure 7:**
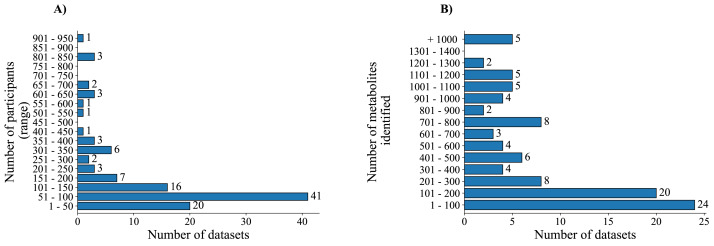
Characterization of shared COVID-19 metabolomics datasets. (A) Distribution of study cohort sizes (number of participants). (B) Number of annotated metabolites per dataset. Data for (B) were manually extracted from repository files in cases where publications lacked explicit reporting.

Figure 7A illustrates the distribution of participant cohort sizes, ranging from 9 to 932 individuals. Despite this broad range, large-scale studies were rare. The majority of datasets (70%) featured 150 or less participants, with the most significant concentration in the 51–100 range (*n* = 41). Conversely, 11 datasets included over 500 participants. This significant variability underscores the diversity of study designs and recruitment capacities, highlighting the need for consistent participant reporting to facilitate cross-study integration and comparability.

This prevalence of small sample sizes raises significant concerns for ML applications. Models trained on limited data risk reduced statistical power, overfitting, and poor external validity, constraining their clinical translation. Consequently, advancing robust, ML-driven COVID-19 research requires a critical shift toward larger, well-characterized metabolomics cohorts.

#### Number of annotated metabolites

Metabolite coverage and annotation varied considerably across the 110 datasets, reflecting diverse analytical strategies: 63 employed untargeted metabolomics approaches, characterized by broad-spectrum profiling without a predefined metabolite panel, while 42 used targeted analyses focusing on specific known compounds, and five adopted a semi-targeted strategy. These methodological differences are essential for interpreting both the breadth of metabolite coverage and the reliability of compound identification.

While 100 datasets reported some form of metabolite annotation—ranging from 3 to 2,732 compounds (Fig. 7B)—adherence to the MSI levels of confidence was low. MSI recommends reporting levels from 1 (confirmed by standards) to 4 (unknowns); however, most datasets omitted these details. Only a small subset explicitly stated their annotations were putative (MSI Levels 2 or 3), based solely on spectral database comparisons (Saito et al., 2024; Overmyer et al., 2021).

#### Geographical region

**Figure 8:**
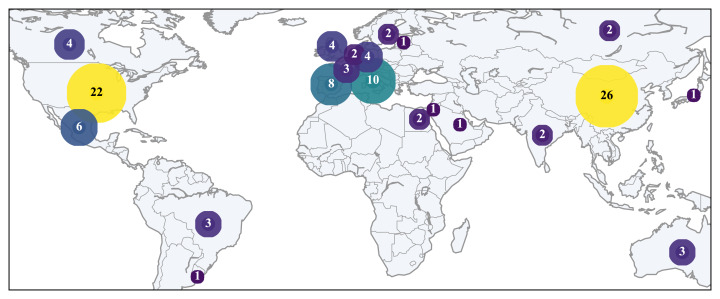
Geographic distribution of dataset contributions by country. Values represent the number of datasets originating from each location.

The 110 datasets originate from 23 countries, showing a broad but uneven global distribution (Fig. 8). The United States (*n* = 22) and China (*n* = 26) are the primary contributors, together accounting for nearly half of all shared data. Detailed counts by country are provided in Table S2.

This regional provenance is critical for data integration, as environmental exposures, diet, genetics, and healthcare systems significantly influence metabolic profiles. Such contextual factors should be considered when comparing results across populations or when seeking to identify robust biomarkers. Furthermore, geographic diversity highlights region-specific metabolic responses, emphasizing the need for transparent reporting of collection contexts to support personalized medicine and global research collaboration.

#### Sample collection periods and temporal coverage

**Figure 9:**
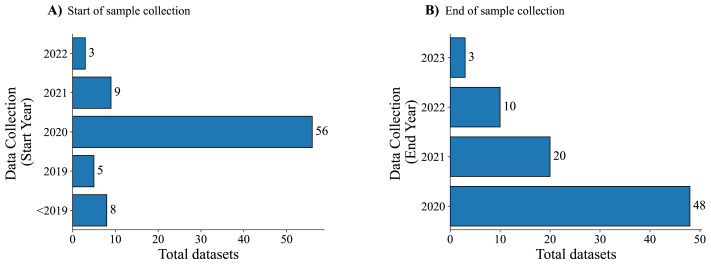
Distribution of sample collection timeframes across COVID-19 metabolomics datasets: (A) start and (B) end of biological sample collection.

Sample collection timeframes varied according to study design, ranging from 1-month windows to multi-year periods. Of the 110 datasets, only 81 (73.6%) explicitly reported collection dates (Fig. 9). Most research peaked during the early pandemic; 48 datasets concluded collection in 2020, followed by 20 in 2021, 10 in 2022 and three during 2023. No datasets reported collection beyond 2023, indicating a recent decline in new COVID-19 metabolomics data production.

Longitudinal studies investigating post-acute sequelae of COVID-19 (PASC) required extended periods for repeated measurements. Examples include studies by Saito et al. (2024), López-Hernández et al. (2023a), Kovarik et al. (2023), Yang et al. (2022), Jing et al. (2022), Zheng et al. (2021), Su et al. (2022), López-Hernández et al. (2023b) and Ozonoff et al. (2024), which collected samples at multiple time points to track metabolic recovery or persistent dysregulation. In contrast, several studies performed retrospective analyses using pre-existing biobank samples collected prior to the COVID-19 pandemic (eight collected before 2019 and five during 2019).

### Q3. Complementary participant data shared and classification factors

#### Additional participant data shared

The reusability and interpretability of metabolomics datasets depend heavily on participant-level metadata—such as sociodemographics, clinical records, and classification criteria—which are essential for stratified modeling and ML. However, while these variables are often summarized in manuscripts, they are frequently omitted from downloadable data files due to privacy concerns. This often results in data fragmentation, where metadata are embedded in Supplementary Tables or shared separately without harmonized formats or participant IDs. Such lack of integration hinders reproducibility and limits the scope of in-depth comparative studies.

**Figure 10:**
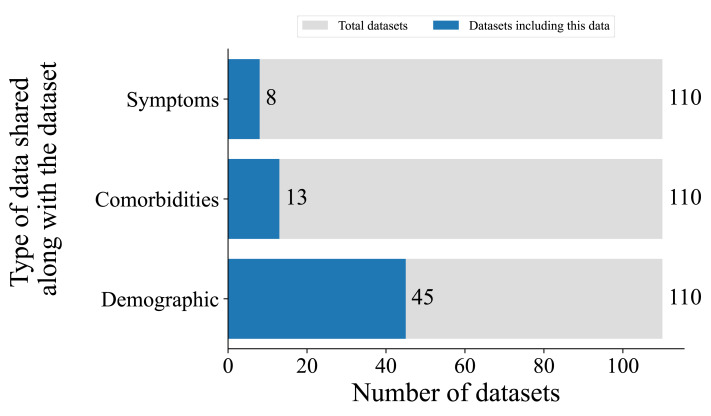
Availability of participant-level metadata across reviewed datasets.

Figure 10 presents a summary of the availability of participant-level metadata across the reviewed datasets. While sociodemographic information was included in 45 datasets (41.8%), only a small proportion reported comorbidity data (11.8%) or symptom records (7.3%). This indicates a systemic gap in metadata completeness, despite the recognized importance of these variables for understanding metabolic alterations in response to COVID-19.

Numerous studies have emphasized that metabolic responses are significantly modulated by individual-level characteristics. For example, Mittelstrass et al. (2011) demonstrated that sex-specific metabolic patterns are prevalent across populations, while Kimhofer et al. (2020) reported that the presence of comorbidities like diabetes and cardiovascular disease significantly alters the metabolic profile and influences COVID-19 severity.

#### Participant classification factors

**Figure 11:**
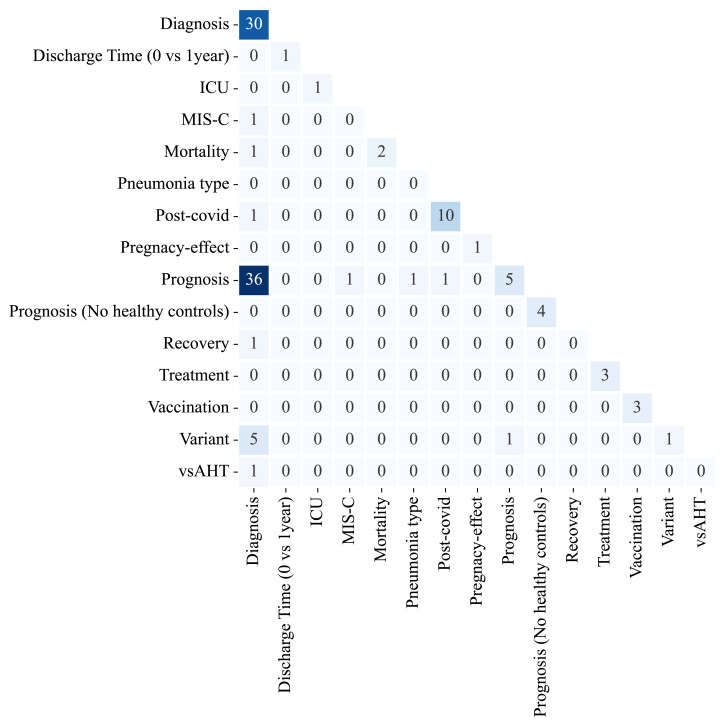
Co-occurrence matrix of participant classification factors used in COVID-19-related metabolomic datasets. Diagonal values represent datasets where the factor was the sole classification variable; off-diagonal values indicate co-occurrence in the same dataset.

The reviewed studies employed diverse and often overlapping classification factors to categorize participants, as illustrated in the co-occurrence matrix (Fig. 11). Diagnosis was the most frequent factor, primarily used to distinguish COVID-19-positive from negative individuals. It frequently co-occurred with Prognosis (36 instances), reflecting a strong research focus on identifying metabolic signatures linked to disease progression and clinical outcomes.

Prognosis also appeared in combination with factors such as post-COVID conditions, mortality, and viral variants, underscoring the use of metabolomics to predict recovery or fatal trajectories. While emerging areas like post-COVID effects (*n* = 11) and the impact of vaccination or treatment (*n* = 3 each) are gaining attention, other clinical variables—including pregnancy, pneumonia type, and comorbidities like arterial hypertension—remain underrepresented, typically appearing in single datasets. This distribution highlights a concentration on acute-phase diagnostics and prognosis, with significant opportunities for expanded research into specific subpopulations and long-term metabolic dysregulation.

Other factors appeared in fewer studies but represent important areas of investigation. Treatment and Vaccination were each reported in three datasets, while Mortality was reported in two datasets, typically in isolation. These variables examine how therapeutic interventions, immune responses, and clinical endpoints influence the metabolome.

Less commonly reported were classification factors such as Variant (one dataset), which explored differences in metabolic profiles associated with different SARS-CoV-2 strains; Pregnancy-effect, examining metabolic alterations in pregnant women with COVID-19; Pneumonia type, which differentiated between viral and bacterial pneumonia manifestations; Discharge Time, used to stratify participants based on the duration of hospitalization; intensive care unit (ICU) admission, distinguishing patients based on whether they required intensive care; and vsAHT (arterial hypertension), which compared individuals with and without pre-existing hypertension. Each of these appeared in only one dataset and typically focused on specific subpopulations or clinical contexts.

## Discussion

Our review reveals a substantial effort in sharing metabolomics data during the COVID-19 pandemic, with nearly three-quarters of studies making datasets available directly or by author request (*n* = 192). This trend reflects a growing recognition of transparency and collaboration during global health crises. However, availability and accessibility remains limited; while about half (*n* = 110) were directly downloadable, a considerable amount required author requests (*n* = 82)—a barrier that reduces efficiency and reproducibility. While protecting patient confidentiality is crucial—particularly given that clinical metabolomics involves sensitive phenotype-associated metadata—this caution often hinders reproducibility and the development of large-scale ML models. The reliance on “data available upon request” is increasingly viewed as insufficient, as response rates are often low and decline over time (Vines et al., 2014). To mitigate privacy risks without compromising open science, researchers should adopt rigorous de-identification protocols (*e.g.*, removing direct identifiers and generalizing sensitive demographic attributes) prior to submission. Furthermore, for highly sensitive clinical data that cannot be fully anonymized, the use of “controlled-access” repositories is a viable strategy. Platforms such as the European Genome-phenome Archive (EGA) or specific sections of MetaboLights allow data to be archived securely, granting access only to approved researchers under Data Use Agreements (DUAs), thereby balancing ethical compliance with the scientific imperative of data reuse (Regulatory, Ethics Working Group & Sugano, 2014; Dyke et al., 2018).

Although specialized repositories provide FAIR-compliant platforms, many datasets were instead deposited as Supplementary Materials or in less structured formats, which complicates discoverability and long-term reuse. These findings suggest that while the community’s openness is commendable, future efforts must prioritize consistent repository deposition, standardized formats, and persistent identifiers to ensure data utility beyond the pandemic’s urgency.

A second key question concerned the data collection methodologies and the characteristics of the datasets. Most studies relied on plasma or serum samples analyzed by LC-MS, GC-MS, or NMR platforms, reflecting methodological diversity but also a lack of harmonization. Dataset sizes ranged from exploratory cohorts with fewer than 50 participants to larger case-control studies, indicating considerable heterogeneity in statistical power. Importantly, most datasets were generated during the first year of the pandemic, with nearly two-thirds concluding by 2020 and none extending beyond 2023. This temporal concentration limits our ability to evaluate metabolic changes associated with emerging variants, reinfections, or long COVID, and underscores the absence of longitudinal designs. Furthermore, reporting practices often failed to include essential details such as confidence levels in metabolite identifications or standardized preprocessing pipelines. Such omissions reduce reproducibility and hinder integrative analyses, revealing that urgency sometimes outweighed methodological rigor.

With regard to our third question, our review found substantial shortcomings. Core participant information—including age, sex, comorbidities, treatment history, and clinical symptoms—was frequently incomplete or absent. These omissions are problematic, as such factors are known to influence metabolic profiles and are critical for stratification analyses. Similarly, classification variables such as disease severity, hospitalization status, or recovery outcomes were often inconsistently defined, making it difficult to compare results across studies or conduct meta-analyses. The absence of longitudinal metadata—including follow-up time and repeated sampling—further limits insights into the dynamics of metabolic changes during infection and recovery. Without comprehensive and standardized metadata, the translational potential of these datasets is significantly compromised.

Together, these findings indicate that while COVID-19 metabolomics studies successfully shared valuable data, substantial challenges remain. The lack of harmonized practices in accessibility, methodological reporting, and metadata collection undermines the long-term utility of these resources. Overcoming these obstacles requires both stronger adherence to FAIR and MSI standards and community-wide initiatives to incentivize consistent metadata reporting and the use of open formats. Addressing these issues will unlock the full potential of COVID-19 datasets, enabling future discoveries through integrative, reproducible, and clinically relevant analyses.

Despite its strengths, several limitations of this review must be acknowledged. First, although our search strategy, while comprehensive, was limited to a specific set of scientific databases and repositories; additional relevant datasets may exist in other platforms, preprints, or specialized data hostings that were not explored. Second, studies published under closed-access conditions were excluded, which may have introduced a bias by omitting potentially valuable datasets. Third, while a second reviewer verified data extraction to minimize errors, a blinded double-extraction process was not performed; thus, inter-rater reliability (*e.g.*, Cohen’s Kappa) was not formally calculated. Furthermore, we did not systematically assess all variables recommended by the MSI, focusing instead on those most consistently reported across studies. Consequently, details regarding quality control procedures, identification confidence scores, or specific preprocessing pipelines may not have been fully captured. Finally, this review did not explore the legal complexities of copyrights or licensing terms governing dataset reuse.

Collectively, these limitations suggest that our findings should be interpreted with caution, while also highlighting important directions for more exhaustive and standardized reviews in the future.

## Conclusions

This systematic review highlights both the achievements and persistent challenges in COVID-19 metabolomics data sharing. Nearly 75% of studies made their datasets available, reflecting a commendable commitment to transparency and collaboration. These shared resources offer significant opportunities for biomarker discovery, integrative analyses, and the application of machine learning.

However, the full utility of these datasets is constrained by non-standardized sharing channels, incomplete participant metadata, and the use of proprietary formats. Inconsistent preprocessing, lack of metabolite identification confidence scores, and heterogeneous classification variables further compromise reproducibility. Furthermore, the concentration of data from the pandemic’s early stages leaves critical areas—such as emerging viral variants and long COVID—underrepresented.

Despite these challenges, the potential for re-analysis, meta-analysis, and integrative studies remains high. Realizing this potential will require the metabolomics community to prioritize standardized metadata reporting, adopt open and FAIR-compliant repositories, and sustain longitudinal data collection. By strengthening data stewardship and methodological harmonization, the field can maximize the long-term impact of COVID-19 metabolomics datasets and ensure that the significant investments made during the pandemic continue to drive meaningful scientific discovery.

## Supplemental Information

10.7717/peerj.21313/supp-1 Supplemental Information 1 Prisma_checklist

10.7717/peerj.21313/supp-2 Supplemental Information 2 Descriptive variables extracted from COVID-19 metabolomic datasets The variables describe key characteristics of each dataset, including study metadata, sample characteristics, analytical platforms, and information related to data availability and accessibility.

10.7717/peerj.21313/supp-3 Supplemental Information 3 Geographic distribution of datasets included in the study. Number of metabolomics datasets included in this systematic review grouped by geographic region according to the country where the study was conducted.

## Additional Information and Declarations

### Data Availability

The following information was supplied regarding data availability: This is a Systematic review/Meta-analysis.
